# Costoclavicular Brachial Plexus Block Facilitates Painless Upper Extremity Reduction: A Case Report

**DOI:** 10.5811/cpcem.59091

**Published:** 2023-09-29

**Authors:** M. Townsend Reeves, Katherine O’Neil, Todd L. Slesinger

**Affiliations:** HCA Florida Aventura Hospital, Department of Emergency Medicine, Aventura, Florida

**Keywords:** costoclavicular brachial plexus block, ultrasound-guided nerve blocks, regional anesthesia, upper extremity, case report

## Abstract

**Introduction:**

The costoclavicular brachial plexus block (CCBPB) has emerged as a more effective approach to regional anesthesia of the upper extremity. The costoclavicular space is the anterior portion of the superior thoracic aperture, located between the clavicle and first rib. The brachial plexus cords traverse this space clustered together in a superficial location lateral to the axillary artery and share a consistent topographical relationship to one another. By targeting the brachial plexus at this specific anatomical location, the CCBPB offers a powerful, single-shot, sensorimotor block of the upper extremity below the shoulder. We present a novel application of the CCBPB to facilitate emergency department (ED) analgesia and closed reduction of an upper extremity fracture.

**Case Report:**

A 25-year-old male presented to the ED with a traumatic Colles fracture sustained during a high-speed motor vehicle collision. Despite multimodal analgesia, the patient reported intractable severe pain with intolerance of radial manipulation. An ultrasound-guided CCBPB was performed to augment pain control and avoid procedural sedation, resulting in dense, surgical anesthesia of the upper extremity, and painless fracture reduction.

**Conclusion:**

Regional anesthesia is an effective component of multimodal pain management and another tool in the emergency physician’s analgesic armamentarium. In acute orthopedic traumas necessitating emergent reduction, regional blocks serve as rescue pain control and can obviate the need for procedural sedation. In terms of targeted upper extremity analgesia, the CCBPB offers effective, single-shot, sensorimotor blockade below the shoulder, mitigating use of opioids and their deleterious side effects, while simultaneously avoiding incomplete blockade or phrenic nerve palsy associated with other approaches to brachial plexus blockade.

CPC-EM Capsule What do we already know about this clinical entity?

*The costoclavicular brachial plexus block (CCBPB) is a novel infraclavicular approach used for perioperative analgesia of the upper extremity.*

What makes this presentation reportable?

*Brachial plexus blockade is rarely used in the emergency department (ED) due to feared complications. The CCBPB is a safe and potent analgesic modality for upper extremity trauma.*

What is the major learning point?

*The CCBPB provides near-complete anesthesia of the upper extremity, mitigating opioid use while avoiding complications associated with other approaches.*

How might this improve emergency medicine practice?

*Regional anesthesia provides a safe, non-euphorigenic, and effective alternative to opioids and procedural sedation for acute pain management in the ED.*

## INTRODUCTION

Nonfatal traumatic injuries account for approximately 30 million emergency department (ED) visits annually in the United States, with acute pain being the most common symptom reported by over 90% of trauma patients.^1^ ^,^ ^2^ Historically, oligoanalgesia has been a concern in emergency pain management.^3^ Given the prevalence and patient burden of post-traumatic pain, emergency physicians should use a multimodal analgesia approach to effectively combat pain and prevent oligoanalgesia. Regional anesthesia is a vital component of multimodal analgesia and is most commonly used in the ED for fracture pain management.^4^ It has been shown to provide excellent pain control, reduce opioid intake and opioid-related side effects (sedation, delirium, pruritus, nausea/vomiting, hypotension), improve post-surgical functional outcomes, shorten hospital length of stay, and possibly reduce the incidence and severity of chronic post-traumatic pain syndrome.^5^

For regional anesthesia of the upper extremity, brachial plexus blockade is the gold standard.^6^ ^,^ ^7^ The ultrasound-guided costoclavicular brachial plexus block (CCBPB) is a novel infraclavicular technique that targets the brachial plexus cords at a superficial, compact, and topographically consistent location, resulting in rapid and reliable upper extremity anesthesia below the shoulder.^8^ The CCBPB provides a complete distribution of anesthesia for the upper extremity, aside from the skin overlying the medial upper arm, which is innervated by the intercostobrachial nerve. In comparison to other approaches to brachial plexus blockade, the CCBPB is a single-shot, low-volume (15–20 milliliters) technique that provides faster onset of sensory blockade compared to the classic lateral sagittal infraclavicular approach, and it results in a lower incidence of hemidiaphragmatic paralysis compared to the supraclavicular approach.^9^ ^,^ ^10^

Although use of the ultrasound-guided brachial plexus blockade in the ED remains a relatively uncommon practice, the CCBPB has the potential for widespread adoption given its ease of performance, rapid onset of analgesia, and low risk of complications.^4^ We present a case report of a patient with intractable upper extremity pain precluding manipulation of a Colles fracture, who underwent ultrasound-guided CCBPB with complete resolution of pain and successful fracture reduction.

## CASE REPORT

A 25-year-old male with no pertinent past medical history presented to the ED by ambulance with obvious right wrist deformity following a high-speed motor vehicle collision as a restrained driver. The patient had braced his outstretched hand against the steering wheel during vehicular impact, resulting in traumatic injury to the wrist. His vital signs on arrival were within normal limits, but he was in significant distress, rating his pain as a 10/10. Physical examination revealed a grossly deformed right wrist with dorsal swelling and severe tenderness to palpation, but no evidence of neurovascular compromise. Radiographs of his right wrist revealed a transverse fracture of the distal radius with dorsal angulation of the distal fragment (Colles fracture), as well as an ulnar styloid fracture.

**Image 1.**
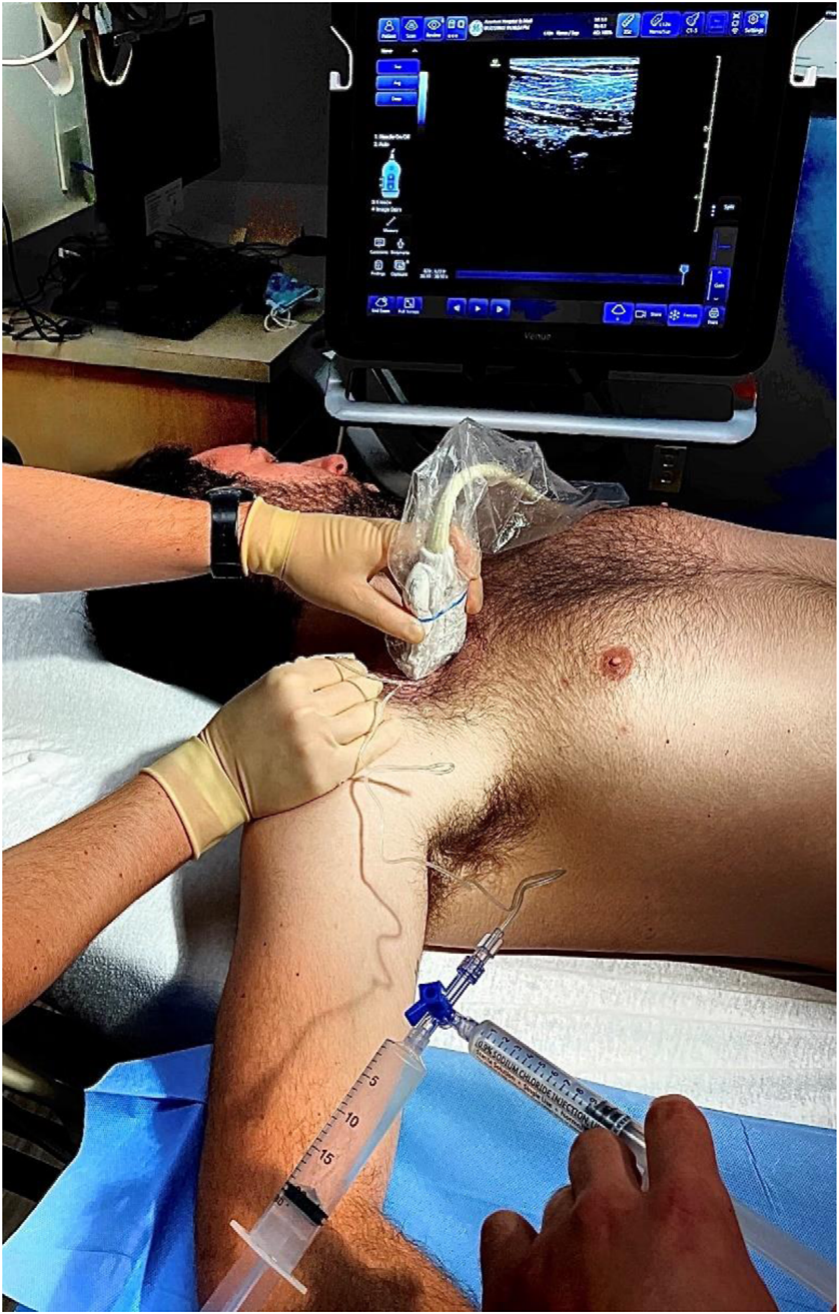
Right-sided costoclavicular brachial plexus block performance. The patient is positioned supine with ipsilateral arm abducted 90 degrees. The high-frequency linear array probe is positioned transversely just below the midpoint of the clavicle in the infraclavicular fossa. The probe beam is angled slightly cephalad to visualize the costoclavicular space posterior to the clavicle. The block needle is inserted lateral-to-medial using a standard in-plane technique. A two-person block technique is used, whereby one clinician performs dynamic needle guidance under ultrasound while the other administers local anesthetic.

**Image 2.**
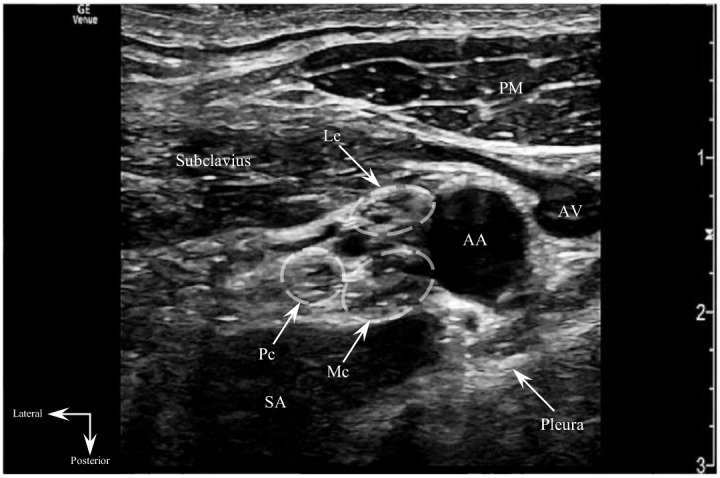
Transverse sonogram of the costoclavicular space depicting the relevant sonoanatomy for costoclavicular brachial plexus block performance. The brachial plexus cords (dashed ovals) are shown lying lateral to the axillary artery and between the intermuscular plane composed of the subclavius and serratus anterior (upper slips) muscles. Note the cords are tightly clustered in a relatively superficial location lateral to the axillary vessels, making the costoclavicular space an ideal target site for brachial plexus blockade. *AA*, axillary artery; *AV*, axillary vein; *Lc*, lateral cord; *Mc*, medial cord; *Pc*, posterior cord; *PM*, pectoralis major; *SA*, serratus anterior.

**Image 3.**
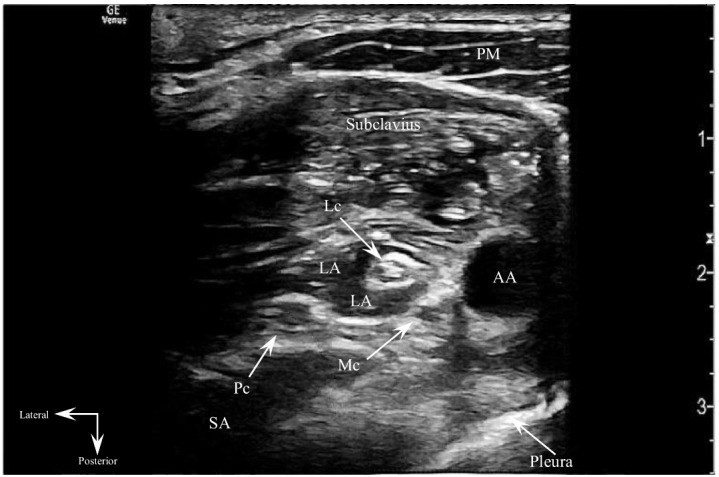
Transverse sonogram post-costoclavicular brachial plexus block performance. Anechoic local anesthetic injectate is visualized enveloping and spreading apart the various costoclavicular brachial plexus cords. *AA*, axillary artery; *LA*, local anesthetic; *Lc*, lateral cord; *Mc*, medial cord; *Pc*, posterior cord; *PM*, pectoralis major; *SA*, serratus anterior.

Despite receiving opioid and nonsteroidal anti-inflammatory analgesia, the patient still reported intractable, severe pain with intolerance of radial manipulation. To avoid procedural sedation, an ultrasound-guided CCBPB was performed to augment patient analgesia and facilitate reduction (Images 1–3, Video).

Informed consent for CCBPB performance was obtained, and the patient was placed on a cardiac monitor with intravenous access established. The patient was positioned supine with the right arm abducted 90 degrees to stretch the pectoralis muscles and bring the costoclavicular brachial plexus more superficial toward the skin surface. The patient was prepped and draped in the standard manner, and sterility was maintained for the duration of the procedure. A high-frequency linear ultrasound probe was oriented transversely just inferior to the midpoint of the right clavicle in the infraclavicular fossa. The probe beam was angled slightly cephalad to visualize the costoclavicular space posterior to the clavicle. The costoclavicular brachial plexus cords were identified just lateral to the axillary artery and between the subclavius and serratus anterior (upper slips) muscles. An in-plane, lateral-to-medial approach was used to guide a 22-gauge, 50-millimeter echogenic block needle between the lateral and posterior cords. Twenty milliliters of 0.5% ropivacaine were injected perineurally around the brachial plexus cords. The procedure was performed without complications.

Fifteen minutes post-block performance, the patient had dense sensorimotor blockade of the right upper extremity and reported his pain at 0/10. Despite aggressive manipulation, the patient reported no pain, and successful reduction of the distal radius fracture was achieved. A sugar-tong forearm splint was applied, and the patient was discharged from the ED with outpatient orthopedic surgery follow-up. On telephone inquiry the next day, the patient reported no numbness, tingling, or residual sensorimotor deficit.

## DISCUSSION

Trauma patients present to the ED at the peak of their pain severity on the trauma care continuum, necessitating analgesic expertise on the part of emergency physicians to effectively treat acute pain. In the wake of the opioid epidemic, there is a dire need for effective yet non-euphorigenic pain management modalities. Regional anesthesia is an ideal peri-traumatic analgesic as it offers targeted, superior pain relief by inhibiting nociceptive signaling of C and Aδ fibers of peripheral nerves, without disruptions in hemodynamics or respiratory status.^11^ Furthermore, it mitigates opioid use, preserves mental status, reduces pain from distracting injuries to enable further assessment of concomitant injuries, and may improve tissue viability by augmenting blood flow to injured tissue via regional sympathectomy.^5^ Long-term effects of early peripheral nerve blockade include improved post-surgical outcomes, greater patient participation in aggressive physical therapy, and possible reduction in incidence or severity of chronic pain syndromes such as complex regional pain syndrome or phantom limb pain.^5^

Regional anesthesia of the upper extremity has historically centered around brachial plexus blockade.^12^ Traditionally, a paracoracoid (lateral sagittal) approach is performed for upper extremity anesthesia in the lateral infraclavicular fossa. However, the brachial plexus cords at this location are deep, diverge from one another, exhibit vast variation in respective location relative to the axillary artery, and are rarely all visualized in one sonographic cross-section.^13^ ^,^ ^14^ At the costoclavicular space (CCS), the brachial plexus cords are more superficial, and clustered together in a consistent anatomical topography lateral to the axillary artery.^15^

The advantageous properties of the costoclavicular brachial plexus allow for shallower needle angles and enhanced needle visualization under ultrasound, with low risk of inadvertent neurovascular or pleural puncture.^16^ Furthermore, faster onset of complete sensory blockade is achieved using lower volumes of local anesthetic compared to the paracoracoid approach, likely due to shorter intercordal diffusion distances at the CCS.^9^ A costoclavicular block dynamics study in which 20 milliliters of 0.5% ropivacaine was used showed median onset time for sensorimotor blockade of five minutes, with complete blockade development within 30 minutes, consistent with our findings.^17^ The rapid onset of analgesia afforded by the CCBPB ultimately obviated the need for procedural sedation, a time-consuming, resource-intensive procedure with rare but significant risk of serious adverse events.

In this case, a single-injection block between the three cords was performed in accordance with the original block description by Karmakar et al.^8^ However, Monzo et al performed a clinical and microanatomical study demonstrating the existence of a reliable intraplexal fascial septum separating the costoclavicular brachial plexus into a superficial compartment comprised of the lateral cord and a deep compartment containing the medial and posterior cords.^18^ They recommend performing a second injection after piercing this intraplexal septum to ensure adequate spread of local anesthetic to the medial and posterior cords, thereby reducing the occurrence of incomplete upper extremity blockade.^18^ Layera et al compared single- vs double-injection CCBPBs, with the double-injection technique resulting in shorter onset and longer duration of anesthesia, although these results were not likely clinically significant.^19^

Our patient exhibited complete pain relief with performance of a single-injection CCBPB. It remains unclear what impact an intraplexal septum plays in local anesthetic diffusion, block onset time, and block success. Further studies are needed to elucidate the optimal CCBPB injection location, clinical significance of intraplexal septa, and the potential need for a multi-injection approach to prevent impedance of local anesthetic spread and subsequent block failure.

Procedural performance time for an ultrasound-guided CCBPB varies depending on multiple factors, including emergency physician proficiency in regional anesthesia, efficiency in patient and procedural setup, and availability of regional block materials. The CCBPB in this case was performed in 15–20 minutes using a two-clinician technique, whereby one performed dynamic needle guidance under ultrasound while the other administered local anesthetic through an attached catheter. Time of performance can be significantly mitigated by having pre-arranged “block bags” or nerve block procedural trays with all materials needed for performance readily available. While a two-clinician technique is not mandatory, it is preferred because it allows for initial tissue hydrodissection with normal saline to confirm needle tip position and open the target injection space prior to deposition of local anesthetic.

Twenty milliliters of 0.5% ropivacaine were used in block performance. In a dose-finding study by Wong et al, this represented the minimum effective volume of ropivacaine in 90% of patients to produce surgical anesthesia after undergoing ultrasound-guided CCBPB.^20^ While this block regimen produced potent analgesia facilitating fracture reduction, the patient’s recovery was delayed post-procedurally given the long-acting properties of ropivacaine. Rapid onset, short-acting local anesthetic (eg, 2% chloroprocaine) may be a more appropriate selection for trauma patients who must undergo painful procedural performance before planned discharge disposition, to ensure restoration of sensorimotor function prior to discharge. While complete restoration of upper extremity sensorimotor function is not a necessity prior to discharge, all patients who undergo brachial plexus blockade should be placed in a shoulder sling and given instructions detailing expected block duration, specialty follow-up, and return precautions in the rare case of persistent sensorimotor deficit.

Performance of the CCBPB is safe, with procedural complications being rare.^16^ The traditional adverse effects of more proximal brachial plexus blockade (Horner syndrome, hemidiaphragmatic paralysis, neuraxial blockade, pneumothorax, hoarseness) are exceedingly uncommon.^9^ ^,^ ^10^ A cadaveric study using methylene blue injections demonstrated consistent sparing of the phrenic nerve, highlighting the preservation of respiratory function post-block performance.^21^ Another cadaveric study analyzing critical structures encountered in the needle trajectory demonstrated no occurrence of vascular puncture, although those authors observed consistent contact of the block needle with the lateral cord.^16^ Use of echogenic block needles, nerve stimulation, and pressure-injection monitoring, as well as block performance on alert patients who can report occurrence of paresthesia, are all safety measures that reduce the incidence of inadvertent neurovascular puncture or injection.^22^ Lastly, emergency physicians should be cognizant of local anesthetic systemic toxicity and adhere to maximum, weight-based, local anesthetic dosing to minimize the occurrence of this potentially fatal clinical entity.

Despite its safety and effectiveness, there are a few limitations of CCBPB performance. Lack of emergency physician training and experience in ultrasound-guided regional anesthesia is a significant limiting factor, as is lack of access to the materials for proper regional block performance. In addition, CCBPB performance may be precluded in certain patient populations, such as the morbidly obese and those with prior history of clavicular trauma, breast surgery, radiotherapy, or mastectomy, due to poor visualization or distortion of the costoclavicular space.

## CONCLUSION

Regional block performance is becoming progressively more ubiquitous in emergency medicine practice. Use of ultrasound-guided nerve blocks by emergency physicians greatly facilitates post-traumatic analgesia and improves long-term patient outcomes. Concerning targeted upper extremity analgesia, the costoclavicular brachial plexus block offers rapid and effective sensorimotor blockade below the level of the shoulder, while avoiding the potential complications associated with opioid utilization or other regional approaches to brachial plexus blockade. Future research is warranted comparing the CCBPB to other upper extremity regional blocks, particularly as it pertains to ED performance in trauma patients.

## Supplementary Information

Video Sonoclip demonstrating costoclavicular brachial plexus block performance. An echogenic block needle is positioned at the injection target site, between the posterior and lateral cords. Anechoic local anesthetic injectate is visualized spreading apart the costoclavicular brachial plexus cords. The lateral cord is completely enveloped in this sonoclip, illustrating the characteristic “donut sign” of regional anesthesia blocks.
*AA*, axillary artery; *LA*, local anesthetic; *Lc*, lateral cord; *Mc*, medial cord; *Pc*, posterior cord; *PM*, pectoralis major; *SA*, serratus anterior.
